# Glioblastoma: Overview of Proteomic Investigations and Biobank Approaches for the Development of a Multidisciplinary Translational Network

**DOI:** 10.3390/cancers17132151

**Published:** 2025-06-26

**Authors:** Giusy Ciuffreda, Sara Casati, Francesca Brambilla, Mauro Campello, Valentina De Falco, Dario Di Silvestre, Antonio Frigeri, Marco Locatelli, Lorenzo Magrassi, Andrea Salmaggi, Marco Salvetti, Francesco Signorelli, Yvan Torrente, Giuseppe Emanuele Umana, Raffaello Viganò, Pietro Luigi Mauri

**Affiliations:** 1 Institute of Biomedical Technologies National Research Council (ITB-CNR), 20054 Segrate, Italy; giusy.ciuffreda@itb.cnr.it (G.C.); francesca.brambilla@itb.cnr.it (F.B.); dario.disilvestre@itb.cnr.it (D.D.S.); raffaello.vigano@itb.cnr.it (R.V.); 2 Institute of Endotypes in Oncology Metabolism and Immunology “G. Salvatore” (IEOMI-CNR), 80131 Naples, Italy or elsi@bbmri.it (S.C.); valentina.defalco@cnr.it (V.D.F.); 3 Common Service ELSI, BBMRI.it—Biobanche e Risorse Biomolecolari, 20126 Milan, Italy; 4 Department of Neuroscience Great Metropolitan Hospital, 89124 Reggio Calabria, Italy; campello.mauro@gmail.com; 5 UniCamillus-Saint Camillus, International University of Health Sciences, 00131 Rome, Italy; 6 Department of Translational Biomedicine and Neuroscience, School of Medicine, University of Bari Aldo Moro, 70124 Bari, Italy; antonio.frigeri@uniba.it (A.F.); francesco.signorelli@uniba.it (F.S.); 7 Neurosurgery Unit, University Hospital Policlinico of Bari, School of Medicine, University of Bari Aldo Moro, 70124 Bari, Italy; 8 Dino Ferrari Center, Department of Pathophysiology and Transplantation, University of Milan, 20122 Milan, Italy; marco.locatelli@policlinico.mi.it (M.L.); yvan.torrente@unimi.it (Y.T.); 9 Division of Neurosurgery, Fondazione IRCCS Ca’ Granda Ospedale Maggiore Policlinico, 20122 Milan, Italy; 10 Neurosurgery, Dipartimento di Scienze Clinico-Chirurgiche e Pediatriche, Università degli Studi di Pavia, Fondazione IRCCS Policlinico S. Matteo, 27100 Pavia, Italy; lorenzo.magrassi@unipv.it; 11 Department of Neurosciences, Azienda Socio-Sanitaria Territoriale (ASST) di Lecco, Ospedale Alessandro Manzoni, 23900 Lecco, Italy; a.salmaggi@asst-lecco.it; 12 Department of Neuroscience, Mental Health and Sensory Organs (NESMOS), Centre for Experimental Neurological Therapies (CENTERS), Sapienza University of Rome, 00185 Rome, Italy; marco.salvetti@uniroma1.it; 13 IRCCS Mediterranean Neurological Institute Neuromed, 86077 Pozzilli, Italy; 14 Neurology Unit, Fondazione IRCCS Ca’ Granda Ospedale Maggiore Policlinico, 20122 Milan, Italy; 15 Department of Medicine and Surgery, University of Enna “Kore”, 94100 Enna, Italy; giuseppe.umana@unikore.it; 16 Department of Neurosurgery, Trauma Center, Gamma Knife Center, Cannizzaro Hospital, 95126 Catania, Italy

**Keywords:** glioblastoma, glioma, brain tumors, proteomics, mass spectrometry, biomarkers, biobanks, translational network, tumor microenvironment, clinical translation

## Abstract

Glioblastoma is a highly aggressive, infiltrative brain tumor of the central nervous system (CNS). Its extensive molecular and biochemical heterogenicity hinders the identification of reliable biomarkers and therapeutic targets, thereby making prognosis and existing therapy ineffective. In recent years, breakthroughs in the use of proteomics on a range of biological samples, such as plasma, cerebrospinal fluid (CSF), tissues, brain cells, and exosomes, represent a potential improvement to GBM investigations. Mass spectrometry-based approaches represent an important technique in the characterization of the tumoral proteome, for the identification of differentially expressed proteins, and for studying altered molecular pathways involved in tumor stages. Proteomics studies advance our knowledge about GBM pathogenesis, the discovery of reliable diagnostic and prognostic markers, and therapeutic approaches, also. In this context, for the effective application of proteomics on GBM, it is mandatory to develop a translational network by integrating hospitals, biobanks, and research institutions into a single network, to enable a collaborative approach across disciplines, thereby enabling rapid translation to clinical application of new proteomic insights. Today, high-quality biobanks play a key role in enabling collaborative, ethically compliant research, supporting the effective application of proteomics in glioblastoma studies and the translation of discoveries into clinical practice. This review explores current trends in proteomics and GBM research, highlighting how leveraging biobank infrastructure and fostering institutional cooperation can drive the development of targeted pilot projects to enhance the impact and effectiveness of glioblastoma research.

## Simple Summary

Glioblastoma (GBM) is one of the most aggressive and hardest to treat forms of brain tumor. It is difficult to find effective therapies because of its high genetic and biological complexity. In recent years, several studies have applied innovative proteomic technologies to study the contribution of proteins to GBM to discover biomarkers that can be applied for innovative diagnostic and therapeutic strategies. The present review summarizes recent developments in this area and outlines the importance of bio-preservation in the collection and storage of biological specimens, which are an absolute requirement for translational proteomics studies. Collaboration between hospitals, biobanks, and research centers can accelerate the development of new biomarkers for diagnosis and treatment, with the aim of improving clinical management and finding more effective therapies for GBM patients.

## 1. Introduction

Gliomas are tumors arising from glial cells, which provide structural and metabolic support to neurons in the central nervous system, playing a crucial role in maintaining neural function and activity [1]. According to the WHO classification, gliomas are graded from I to IV based on their histopathological characteristics, reflecting increasing malignancy. Among them, glioblastoma multiforme represents the most aggressive and lethal form (WHO grade IV), accounting for 54% of all gliomas and 50.9% of malignant brain and CNS tumors [2]. Typically diagnosed in individuals between 55 and 80 years old [3], recent evidence suggests that GBM can also develop in children, adolescents, and young adults, although its incidence is lower in younger populations and decreases with decreasing age [4]. Despite intensive therapeutic strategies, including surgical resection, adjuvant radiotherapy, and chemotherapy with temozolomide, GBM remains largely incurable, with a median survival of only 15 months and a 5-year survival rate of 7.2% [5,6,7]. GBM is characterized by rapid proliferation, diffuse infiltration into surrounding brain tissue, and a highly heterogeneous molecular landscape, which contributes to its aggressive behavior and therapy resistance [1,8]. Molecular profiling studies, particularly from The Cancer Genome Atlas (TCGA), have identified key genetic alterations underlying GBM pathogenesis, including EGFR amplification, PTEN deletion, TP53 mutations [9], and IDH1/IDH2 alterations, which are associated with distinct molecular subtypes and clinical outcomes [10]. Epigenetically, O6-methylguanine-DNA methyltransferase (MGMT) promoter methylation is a key biomarker influencing the response to temozolomide (TMZ) chemotherapy, as its silencing prevents DNA repair, increasing susceptibility to alkylating agents [11,12,13]. Additionally, widespread DNA hypermethylation and histone modifications contribute to tumor plasticity and therapeutic resistance [14].

GBM is broadly classified into two major subtypes [15]. Primary GBM is most common in elderly patients (>60 years) and is characterized by EGFR amplification (40–50%), with a subset carrying the EGFRvIII mutation, PTEN loss, CDKN2A deletion, and TERT promoter mutations, which drive rapid proliferation and invasion [16,17,18,19,20]. Secondary GBM occurs more frequently in younger patients (<45 years) [21]. This subtype is strongly associated with IDH1/IDH2 mutations (>80%), which induce a distinct metabolic phenotype by altering α-ketoglutarate production and promoting DNA and histone hypermethylation (the glioma CpG island methylator phenotype, G-CIMP) [22]. Unlike primary GBM, EGFR amplification is rare, while TP53 mutations (65–80%), ATRX, and chromosome 19q loss are more prevalent [23]. The biological distinction between GBM subtypes impacts prognosis and therapy, with IDH-mutant (secondary) GBM showing better outcomes and greater chemosensitivity than IDH-wildtype (primary) GBM [24]. These molecular differences emphasize the need for personalized treatment strategies, and emerging proteomic studies aim to identify protein biomarkers that can differentiate these subtypes at a molecular and functional level. In addition to genetic alterations, GBM shows remarkable heterogeneity at the cellular, metabolic, and microenvironmental levels, which significantly contribute to its aggressive phenotype. Glioblastoma stem-like cells (GSCs), expressing stemness markers (SOX2, Nestin, CD133), have been identified as a key driver of tumor recurrence due to their self-renewal capacity and resistance to conventional therapies largely mediated by Wnt, Notch, and Hedgehog signaling pathways [25]. The hypoxic microenvironment of GBM further promotes tumor progression through hypoxia-inducible factor 1-alpha (HIF-1α) activation and vascular endothelial growth factor (VEGF)-driven angiogenesis, enhancing invasion and limiting therapeutic efficacy [26]. Moreover, GBM is characterized by a highly immunosuppressive environment, with mechanisms such as programmed death-ligand 1 (PD-L1) upregulation, transforming growth factor beta (TGF-β) secretion, and the recruitment of regulatory T cells (Tregs) and tumor-associated macrophages (TAMs), which collectively suppress antitumor immune responses and contribute to resistance to immunotherapy [27,28].

Several critical signaling pathways drive GBM pathogenesis and progression, making it one of the most molecularly complex tumors. The phosphoinositide 3-kinase/protein kinase B/mammalian target of rapamycin (PI3K/AKT/Mtor) pathway is frequently activated in GBM due to PTEN loss or EGFR mutations, promoting cell proliferation, survival, and resistance to apoptosis [29]. Similarly, mutations and amplifications in upstream regulators such as EGFR and platelet-derived growth factor receptor (PDGFR) activate the RAS/MAPK pathway, further enhancing tumor growth and invasion. Another hallmark alteration is the inactivation of p53 via mutations or MDM2 amplification, which allows GBM cells to evade apoptosis and sustain proliferation [30]. Furthermore, dysregulated Wnt signaling has been shown to contribute to glioblastoma stem cell maintenance and therapy resistance, reinforcing the tumor’s ability to persist despite aggressive treatment regimens [31,32]. Hypoxic regions within the tumor promote angiogenesis through VEGF signaling, supporting tumor growth and invasive potential [33,34]. Moreover, GBM cells themselves exhibit extreme invasiveness, allowing them to infiltrate surrounding brain tissue, making complete surgical resection nearly impossible [35]. Additionally, matrix metalloproteinases (MMPs) facilitate extracellular matrix degradation, further promoting tumor cell migration [36,37]. Another key challenge in GBM treatment is its ability to disrupt the blood–brain barrier (BBB), promoting tumor infiltration while simultaneously limiting drug delivery, thereby posing a major obstacle to effective pharmacological intervention [38]. This highly dynamic tumor ecosystem poses a significant challenge to treatment, requiring novel approaches that integrate multi-omics technologies to dissect GBM biology at different regulatory levels.

Although genomic and transcriptomic analyses have provided critical insights into the molecular classification of GBM, these approaches primarily capture static genetic alterations and fail to reflect dynamic molecular changes that occur during tumor progression and treatment. Proteomics has emerged as a powerful tool to address this limitation, allowing for the characterization of protein expression patterns, post-translational modifications, and protein–protein interactions that regulate key tumorigenic processes. Among the most relevant proteomic approaches in recent years in the study of brain tumors, especially glioblastoma, mass spectrometry has emerged as a crucial tool for analyzing the tumoral proteome. With advanced technologies like mass spectrometry, proteomic analysis has highlighted significant alterations in GBM tumors compared to normal brain tissue. Mass spectrometry-based proteomics has indeed allowed the identification of protein signatures associated with GBM pathogenesis, highlighting key alterations in metabolic enzymes, membrane proteins, and immune regulators [39]. Recent studies have demonstrated that GBM tumors exhibit significant overexpression of proteins involved in cellular movement, antigen presentation, and cell–cell signaling compared to normal brain tissue [17,39]. Furthermore, proteomic profiling has distinguished IDH1-mutant from IDH1-wildtype GBM by revealing specific metabolic protein alterations, such as increased expression of aldehyde dehydrogenase 1 family member A3 (ALDH1A3) and IDH1-R132H in IDH-mutant tumors, underscoring the relevance of metabolic reprogramming in gliomagenesis [40]. One of the most promising applications of proteomics lies in liquid biopsy, where the detection of extracellular vesicles (EVs), circulating tumor proteins, and cerebrospinal fluid biomarkers offer a minimally invasive strategy for GBM diagnosis and disease monitoring. Several studies have identified tumor-derived EVs carrying proteins such as CD9, tumor susceptibility gene 101 (TSG101), and heat shock protein 70 (HSP70) as potential biomarkers for GBM classification and prognosis [16,41]. These findings indicate that proteomics has the potential not only to enhance understanding of the pathophysiology of GBM, but also to improve therapeutic processes through biomarker validation and personalized medicine. Achievement of therapy resistance will require an integration of proteomic data with genomic, transcriptomic, and metabolomic approaches to identify the perturbed patient specific critical issues.

Notably, proteomics shares with radiomics the ambitious objective of serving as a diagnostic alternative to histological sampling, an approach of particular relevance for tumors located in eloquent regions such as the brainstem.

This review is focused on current trends in the field of proteomics and GBM research to analyze the role of proteomics in the development of key biomarkers, molecular pathways, and novel therapeutics that can enhance clinical management and improve patient health. It is pursued as part of a broader effort to consolidate the Italian translational network of glioblastoma which includes hospitals, biobanks, and research centers. The objective is to incorporate proteomic evaluation into the framework of translational research, using the infrastructure of high-quality biobanks and fostering a cooperative approach that meets European and international standards. In particular, the network provides the tools to facilitate sharing of biospecimens and associated information, their collection and storage standardization, and the development of focused pilot projects, thus helping to integrate and improve the effectiveness of glioblastoma research.

Building on this infrastructure, the future employment of AI techniques may further enhance the interpretation of proteomic data, with the ultimate goal of achieving a cure for glioblastoma [42].

## 2. GBM Incidence

**Figure 1:**
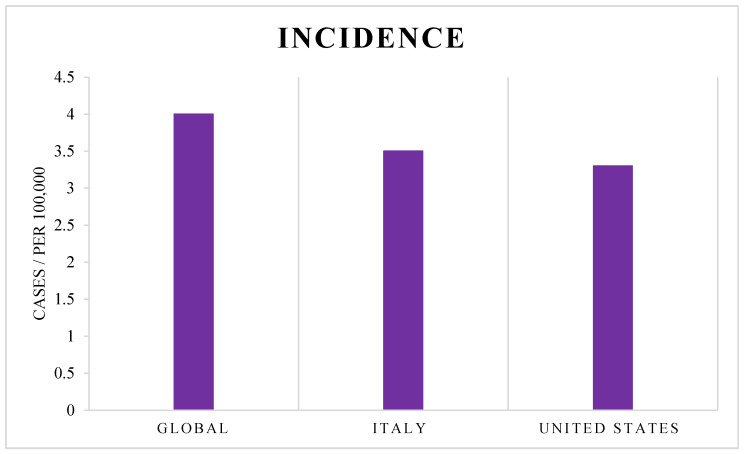
Reported incidence rates of glioblastoma (cases per 100,000 people) globally, in Italy, and in the United States, based on the most recent data from the NCI and ISS.

In terms of GBM incidence, there are substantial variations in the incidence of glioblastoma both at the global level and in specific countries [43,44]. This fact is due to a multitude of combined factors such as genetics, environment, and socioeconomic regions. The National Cancer Institute (NCI, https://www.cancer.gov/, accessed on 24 March 2025) reported that the incidence rate of glioblastoma, approximately 3 to 4 cases per 100,000 people per year, is relatively consistent worldwide. In the United States, the incidence is around 3.3 cases for every 100,000 people (4.07 for males and 2.58 for females), based on a study conducted during the years of 2017 and 2021 [45]. Current statistics from the Istituto Superiore di Sanità (ISS, https://www.iss.it/, Italy, accessed on 24 March 2025) suggest that the incidence of GBM in Italy is approximately 3.5 cases per 100,000 people, a statistic largely supported by the most recent data available [46] (Figure 1).

**Figure 2:**
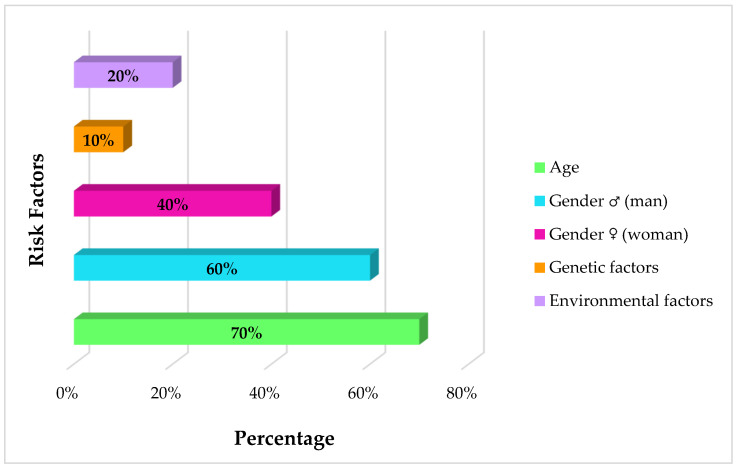
Risk factors for glioblastoma. Stacked bar chart depicting the percentage distribution of age [47], sex [48], environmental exposure [53,54,55], and family history [56] among patients diagnosed with glioblastoma. Note that the risk factors are not mutually exclusive. Data synthesized from studies.

The incidence of glioblastoma has several risk factors associated with it (Figure 2). Firstly, age is an important factor: the incidence is low and increases significantly between the ages of 75 and 84 [47]. This increase is associated with changes in the immune system and inflammatory processes associated with the promotion of tumors in older people. Indeed, aging is accompanied by greater central nervous system inflammation and increased immunosuppressive TGFβ and interleukin 10 (IL-10) factors that impair the immune system and can cause a tumor onset [47].

Moreover, there is also a difference in GBM incidence in relation to sex: men have a greater burden than women (1.6:1) and, possibly, a worse prognosis [48]. These differences make male astrocytes more susceptible to aggressive transformation, characterized by loss of the tumor protein p53 (TP53) tumor suppressor gene and downregulation of RB1 expression, leading to the acquisition of a stem cell-like phenotype with high proliferation and renewal rates [49,50]. In contrast, female cells show higher cyclin-dependent kinase inhibitor 1A (CDKN1A) expression, which helps preserve cell cycle regulation despite TP53 mutations, thus contributing to greater cellular protection [51]. The differences between males and females could also be ascribed to the X chromosome via KDM6A, a tumor suppressor gene which is known to be more expressed in female cells [52].

Environmental aspects, like ionizing radiation from cancer treatments or toxic exposures like diesel smoke and spray paints, are also linked to greater risk, particularly in early age exposure [53,54,55].

In addition, familial history is reported to contribute in 5–10% of glioma cases [56]. The first-degree relatives of glioma patients carry twofold risks of succumbing to brain tumors, particularly if the illness is diagnosed during early years [57]. Inherited Li-Fraumeni syndrome, Turcot syndrome, and Neurofibromatosis type-1 increase risk of GBM and demonstrate how genetic changes in oncogenes and oncosuppressors are plausible mediators of gliomagenesis [47]. Children born to elderly parents or those with certain congenital central nervous system disabilities also have an increased susceptibility to astrocytoma [58,59].

## 3. Proteomics Analysis in Glioblastoma Disease

After emphasis on epidemiology and the major risk factors of glioblastoma, it becomes relevant to detail the various types of biological specimens for proteomes of tumors. Concerning biomarker discovery on Glioblastoma, about 8000 articles can be found on PubMed, including 5500 between 2014 and 2024, spanning a wide range of omics approaches for biomarker identification. Omics approaches have allowed the exploration of different aspects of glioblastoma, from the discovery of relevant genetic mutations to the analysis of differential protein profiles and the characterization of altered metabolites.

**Figure 3:**
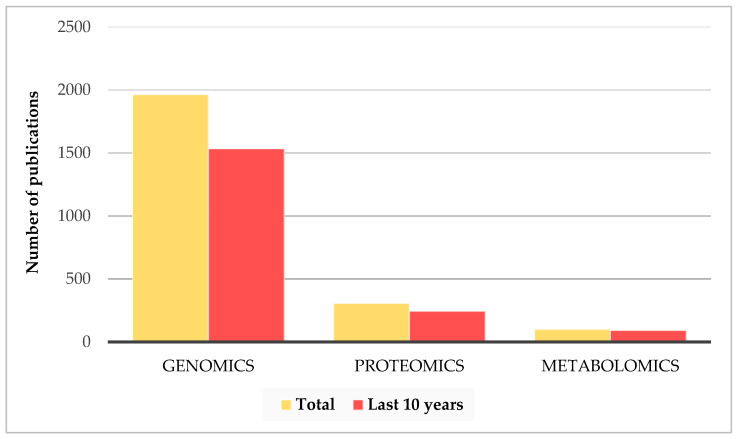
Distribution of PubMed articles on glioblastoma genomics, proteomics, and metabolomics. Yellow bars indicate total publications; red bars indicate publications from the last 10 years. Recent studies account for 78% of genomics, 80% of proteomics, and 91% of metabolomics publications.

Genomics studies represent a significant component of the glioblastoma literature (Figure 3) due to their ability to identify genetic mutations, epigenetic alterations, and changes in gene expression that influence tumor progression. The comparison shown in Figure 3 is based on PubMed searches using the keywords ‘Glioblastoma Proteomics Biomarker’, ‘Glioblastoma Genomics Biomarker’, and ‘Glioblastoma Metabolomics Biomarker’.

Among the various omics approaches, proteomics has become increasingly prominent in glioblastoma research. Approximately 80% of proteomics studies on glioblastoma have been published in the last ten years, with the majority utilizing liquid chromatography coupled with high-resolution tandem mass spectrometry (LC-MS/MS), highlighting the growing interest in this technique. In total, 300 articles focused on the application of proteomics were identified, reflecting the growing interest in these approaches. This approach has shown promise for the identification of novel biomarkers and brought about more detailed understanding of the molecular complexity of glioblastoma.

**Figure 4:**
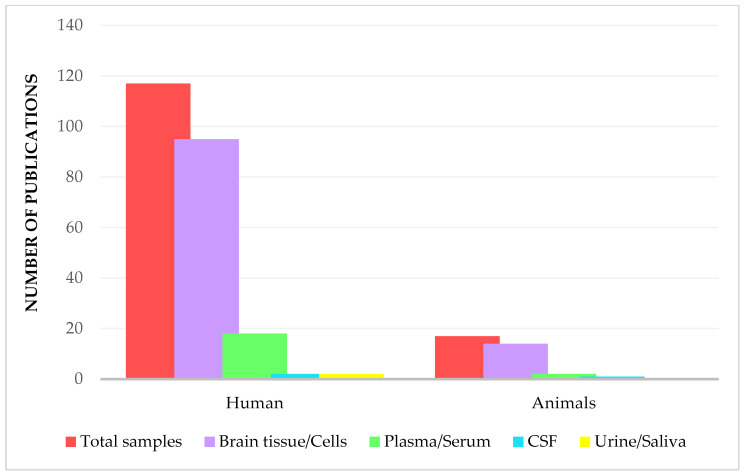
PubMed-based distribution of glioblastoma-related articles (2014–2024), grouped by sample type (brain tissue, CSF, plasma, serum, urine, saliva) and categorized as human or animal studies. The histogram emphasizes the prevalence of tissue-based studies and highlights the relative underrepresentation of biofluid-based research.

In the following sections we will review the main proteomics studies, according to the type of sample analyzed. Notable proteomics investigations on GBM biomarkers were focused on human samples mainly. Importantly, most studies focus on brain tissue, which is considered the main site for molecular characterization of glioblastoma. However, analysis of biofluids such as CSF, plasma, serum, urine, and saliva offers complementary information on proteomic alterations associated with GBM disease (Figure 4). The distribution illustrated in Figure 4 was derived through a meticulous manual curation of PubMed articles, initially identified using broad and specific keyword combinations, including ‘Glioblastoma’, ‘Proteomics’, ‘Biomarker’, and ‘Glioblastoma Proteomics (mass spectrometry)’. Each study was then carefully classified by sample type and species, based on a thorough review rather than just keyword filtering.

In the following sections, we will explore how various types of samples—from brain tissue to biofluids—contribute to our understanding of GBM.

### 3.1. Brain Tissue and Related Cell Lines

The proteomics analysis of glioblastoma is mostly applied to the brain tissue samples, which are the primary biological material for the evaluation of the involved proteins for in this highly malignant neoplasm. The differences and types of tissues collected, whether they are fresh or formalin-fixed, influence the precision of the analyses and affect the biomedical detection and the understanding of the molecular pathobiology of the disease. Besides tissue samples, brain cell lines are repeatedly employed as in vitro models for the analysis of proteomes of glioblastoma. The primary cell cultures are an acceptable, albeit incomplete, way to examine the tumor’s molecular mechanisms in an orchestrated environment that is devoid of the actual complexity and heterogenecity of the tumor.

Table 1 presents the bibliographic quotes of the main publications from the last ten years, indicative of the most notable candidate protein biomarkers, type of samples and/or cell culture used, and the proteomics techniques applied. These protein biomarkers are mostly considered candidate biomarkers identified through proteomic analyses; however, their clinical validation status varies across studies.

Of note, the identified proteins may occur in GBM in the form of different proteoforms, thus requiring further studies for their in-depth characterization.

In recent years, LC-MS/MS has become indispensable to study the glioblastoma proteome, allowing the discovery of proteins that are crucial in tumor progression and therapeutic resistance.

Leveraging LC-MS/MS combined with Data-Independent Acquisition (DIA) across multiple studies, researchers have uncovered key proteins and regulatory mechanisms involved in glioblastoma progression and therapeutic resistance. For example, El-Baba et al. [83] pinpointed tumorigenic proteins, including solute carrier family 2 member 1 (SLC2A1), alongside inhibitory genes like phosphatidylethanolamine-binding protein 1 (PEBP1), emphasizing their influence on cell division and nutrient uptake, vital actions in aggressive brain tumors. Naryzhny et al. [85] identified numerous common proteins in various glioblastoma cell lines, such as annexin A1 (ANXA1), ANXA2, and vimentin (VIME), which play a role in several biological processes essential for tumor progression, including exosome formation, cell adhesion, modulation of the immune response, and various metabolic mechanisms. These proteins are also involved in radioresistance and drug resistance, key phenomena for the survival of tumor cells during radiation therapy. In particular, ANXA1 and VIME, involved in invasiveness and cell adhesion, contribute to glioblastoma’s ability to resist the damaging effects of radiotherapy.

Through proteomic profiling, Azzalin et al. [114] demonstrated that glioblastoma cells, specifically the U-87 MG glioblastoma cell line and other glioma cultures, respond to glucose deprivation by upregulating SHC-transforming protein 3 (SHC3), a neuronal adaptor protein with roles in signal transduction and vesicular trafficking. Elevated SHC3 levels enhanced glucose uptake by promoting the translocation of GLUT/SLC2A transporters to the plasma membrane, sustaining the high glycolytic activity typical of glioblastoma. SHC3-mediated inhibition of poly (ADP-ribose) polymerase 1 (PARP1) was linked to altered trafficking and glycosylation of glucose transporters, indicating a regulatory axis that modulates metabolic adaptation under nutrient stress. These findings identify SHC3 and PARP1 as potential metabolic biomarkers in glioblastoma.

Another study by Hu et al. [86] identified differentially expressed proteins between different glioblastoma cell lines, highlighting the role of proteins such as RRAS, protein tyrosine phosphatase receptor type O (PTPRO), and those involved in the phosphoinositide 3-kinase/protein kinase B (PI3K/AKT) and nuclear factor kappa-light-chain-enhancer of activated B cells (NF-κB) pathways, all crucial for regulating cellular processes that promote tumor progression. Moreover, the study showed how the integration of these pathways influences interactions with the extracellular matrix (ECM), a key element in glioblastoma invasiveness [86]. Menezes et al. [87], focused on biomarkers such as Decorin (DCN) and Glypican-1 (GPC-1), both involved in angiogenesis and tumor growth. The study revealed that the inhibition of histone deacetylases (HDAC) alters the so-called AngioMatrix, a set of proteins and molecules that regulate the tumor’s vascular environment, significantly influencing angiogenesis, cell motility, and tumor progression. DCN, in particular, was identified as a key prognostic biomarker, suggesting that its expression could be correlated with the severity and evolution of glioblastoma.

Another crucial aspect concerns resistance to treatments, particularly TMZ, a drug commonly used to treat glioblastoma. In this regard, Yi et al. [91] identified proteins involved in resistance to TMZ, playing roles in various signaling pathways such as actin cytoskeleton regulation, the PI3K-Akt pathway, and focal adhesion and phagosome signaling. Proteins like DEAH-box helicase 9 (DHX9), heterogeneous nuclear ribonucleoprotein R (HNRNPR), and ribosomal protein L3 (RPL3) influence these processes, altering the ability of tumor cells to adapt and survive under treatment. Focal adhesion pathways are crucial in tumor invasiveness, facilitating cell migration and spread, but also determining the response to chemotherapy. Naryzhny et al. [115] have shown that GBM resistant to standard therapies, such as TMZ, transition to a neuronal-like state. This phenotypic change is associated with multilevel activation of the RAS-mitogen-activated protein kinase (RAS-MAPK) signaling pathway, driven by increased or hyperphosphorylation of key components such as RAS, BRAF, and MAPK.

In parallel, Mallawaaratchy et al. [96,97] focused on extracellular vesicles, identifying proteins such as ANXA1 and Integrin beta-1 (ITGB1) as biomarkers in glioblastoma cell invasiveness. These proteins play not only a role in invasiveness, but they are also involved in processes like actin polymerization and endosomal sorting, essential for invadopodia formation, specialized cellular structures involved in tumor cell migration and invasion. In particular, ANXA1 is known for its role in regulating the cell membrane and modulating inflammatory responses, while ITGB1 is involved in cell adhesion and interactions with the extracellular matrix.

Bijnsdorp et al. [101] analyzed the U87WT (wild-type) and U87vIII (mutant) cell lines, revealing key mechanisms underlying tumor aggressiveness and treatment response. The results highlighted different phosphorylation activities and kinase signaling pathways, such as EGFR, CDK1/2/7, GSK3B, AKT1, MAPK1/3, MET, PAK2/4, and PRKCA/B. In particular, EVs from U87vIII cells (mutant for EGFR) carry active EGFR and other kinases, suggesting that EVs may act as signaling vehicles for transmitting signals between tumor cells and the surrounding microenvironment. Xue et al. [70] analyzed glioblastoma tumor tissues, identifying numerous differentially expressed proteins, including key factors such as PP1γ, YAP1, and SOX2, which emerged as particularly relevant for glioma progression. The study revealed that the Hippo signaling pathway, essential for regulating cell growth and proliferation, is activated in glioblastoma. In particular, PP1γ promotes the nuclear translocation of YAP1, a key event for its oncogenic activity and glioma progression and poor prognosis. Jeon et al. [71] explored biomarkers associated with responses to anti-angiogenic therapies in glioblastoma, identifying key proteins such as TMEM173 and FADD, which regulate immune and apoptotic processes. Additionally, ERCC2 and POLD1, proteins involved in DNA repair mechanisms, were associated with poorer prognosis in glioblastoma patients, suggesting that ERCC2 and POLD1 play a crucial role in the response to DNA damage.

An additional advancement in proteomic technology is SWATH-MS (Sequential Window Acquisition of All Theoretical Mass Spectra). Maire et al. [82] identified 104 differentially expressed proteins in extracellular vesicles derived from glioblastoma cells. Among these, CD44, Integrin-β1, and Tetraspanin-14 were highlighted for their involvement in key oncogenic pathways such as PI3K/AKT and MAPK. CD44 promotes cell adhesion and invasiveness, Integrin-β1 regulates migration and focal adhesion, and Tetraspanin-14 contributes to cytoskeletal remodeling and immune response. These proteins also play roles in DNA repair and apoptosis, ultimately supporting tumor survival and progression. In another study, Schulze et al. [89] analyzed U87 and U251 cell lines, identifying biomarkers such as DAB1 and RELN, crucial for cell migration and survival. RELN, overexpressed in the neural subtype of glioblastoma, is associated with better survival but is often silenced by methylation. The interaction between DAB1 and RELN was seen as crucial for limiting glioma progression. DAB1 tumor-suppressive effect suggests that enhancing its expression or activating the RELN-DAB1 pathway could reduce tumor aggressiveness and improve treatment response.

Zheng et al. [65] analyzed radioresistant glioblastoma cell lines (U251, U251R, T98G) and xenografted tumors using quantitative proteomics with Tandem Mass Tag (TMT), identifying 17 upregulated proteins, among which SDC1 and TGM2 were found to be crucial for radioresistance and poor prognosis. Both proteins support mechanisms that increase treatment resistance and worsen prognosis.

Another study by Cosenza-Contrares et al. [60] analyzed initial and recurrent glioblastoma samples, identifying ASAH1 and GPNMB as key regulators. ASAH1 is crucial for sphingosine metabolism and interaction with tumor-associated macrophages, while GPNMB modulates the extracellular matrix, promoting tumor invasiveness. This study highlighted the role of immune pathways and signaling through interleukins [62]. Oh et al. [63] conducted a proteomic analysis on various glioblastoma subtypes, revealing important metabolic vulnerabilities. Among the key proteins, PHGDH stands out for its ability to support cell proliferation under hypoxic conditions.

Jang et al. [69] highlighted biological differences between male and female glioblastoma patients, noting distinctions in tumor mechanisms. In males, EGFR receptor hyperactivation was observed, associated with more aggressive progression, along with specific biomarkers such as COL28A1 and EDNRB; in females, the protein SPP1 (Osteopontin) was crucial in microenvironmental interactions that favor tumor growth.

Finally, Nikitina et al. [116] examined glioma cell lines (DBTRG-05MG), showing that type I interferon and VSV infections influence the cellular response through EGFR and HER2-mediated pathways. Additionally, the EGFR inhibitor gefitinib showed a synergistic effect with interferon signaling, suggesting potential strategies for antiviral and immunological therapies.

### 3.2. Biofluids

Biofluids and extracellular vesicles have attracted increasing attention as potential sources of biomarkers in GBM. Various biofluids, such as CSF, the fluid fraction obtained through ultrasonic aspiration of brain tumor tissue during neurosurgery (CUSA), serum, and plasma, have been used in proteomic analyses to identify tumor-associated protein markers [117,118,119]. While each of these fluids offers valuable insights into tumor biology, they also come with limitations. For instance, collecting CSF or CUSA fluid is highly invasive and not feasible for routine use. Conversely, peripheral blood is easily accessible but contains a complex mix of molecular components, which can hinder the detection of tumor-specific biomarkers [120]. Table 2 presents a summary of biofluids investigated in proteomic research and the corresponding candidate biomarkers.

#### 3.2.1. Cerebrospinal Fluid

Cerebrospinal fluid is ideal for liquid biopsy in brain tumors because it directly contacts the tumor environment and bypasses the BBB [41], making it less invasive than tissue biopsy [130].

Schmid et al. [121] analyzed the CSF proteome of glioblastoma patients using LC-MS/MS with Data-Dependent Acquisition (DDA) to identify differentially expressed proteins (DEPs). In total, 349 proteins were identified, with more evident alterations in patients with BBB disruption; among these, CHI3L1 and GFAP emerged as promising biomarkers. CHI3L1 was previously associated with neurological diseases such as multiple sclerosis, Alzheimer’s disease [131], and systemic cancers [132]. Additionally, CHI3L1 contributes to tumor aggressiveness by binding to the interleukin-13 receptor (IL13RA2) and inducing a mesenchymal phenotype [133]. GFAP, on the other hand, proved to be a relevant diagnostic biomarker, with higher levels in the CSF of glioblastoma patients compared to other brain tumors and associated with reduced survival [134,135,136].

Of particular interest, CSF proteomic analysis identified two clusters of patients with distinct biological characteristics. The first cluster showed an upregulation of proteins involved in coagulation, such as fibrin (FGA, FGB, FGG) and thrombin (F2), which have previously been associated with the aggressiveness of glioblastoma [121]. This cluster is also characterized by the activation of the “LXR/RXR Activation” pathway, which could represent a therapeutic target. Conversely, the second cluster displays less aggressive features, with pathways associated with synaptogenesis and immunomodulation [41,137,138,139].

A further study by Mikolajewicz et al. [41] detected 755 proteins including GAP43 as a specific biomarker for glioblastoma, while other proteins, such as SERPIN3 and APOE, are involved in pathways related to BBB degradation, angiogenesis, and stemness.

A complementary investigation by Magrassi et al. [122] explored the proteomic composition of cystic fluids from various brain tumors, including secretory meningiomas, cystic schwannomas, and high-grade gliomas. Their study highlights that plasma proteins, such as albumin, haptoglobin, fibrinogen, and transferrin, which leak from disrupted BBB, constitute a significant portion of the cystic fluid proteome and are abundant across all tumor types. Additionally, proteins derived from cerebrospinal fluid, such as prostaglandin D2 synthase, were identified, suggesting a mixed origin of cystic fluid from both plasma and CSF. These findings underscore the potential of cystic fluid proteomics to uncover novel biomarkers and therapeutic targets in brain tumors. Looking ahead, these proteomic signatures could pave the way for minimally invasive diagnostic tools, such as liquid biopsies, which would facilitate earlier detection and personalized treatment strategies.

#### 3.2.2. Plasma

Plasma, in particular, has emerged as a matrix of great interest for proteomic research due to its accessibility and its ability to reflect the physiological and pathological state of the patient. However, the presence of abundant proteins such as albumin and immunoglobulin can obscure low-abundance biomarkers, requiring advanced fractionation and protein depletion techniques to improve the sensitivity of analyses.

The study by Cosenza-Contreras et al. [60] analyzed the proteomic profiles of tumor samples from patients with initial (iGBM) and recurrent (rGBM) glioblastomas. Among the identified proteins, ASAH1, SYNM, and GPNMB were found to be significantly upregulated in rGBM. In particular, ASAH1 was associated with increased neutrophil involvement in the tumor microenvironment, while SYNM was expressed in early tumors compared with healthy controls [27].

Research by Sabbagh et al. [124] placed attention on von Willebrand factor (VWF), identifying it as a possible specific biomarker in plasma EVs of GBM patients, as it may be involved in pro-angiogenic processes and tumor vascularization. The enrichment of VWF in plasma-derived EVs of GBM patients could reflect its participation in the tumor-typical angiogenic and pro-thrombotic response. Finally, the study by Naryzhny et al. [123] investigated plasma proteoforms of haptoglobin (Hp), a key protein in hemoglobin binding, oxidative stress protection, and inflammation control. Hp is an active component of plasma and is involved in numerous processes that are fundamental to homeostasis in the human body. In particular, it binds free hemoglobin (Hb), protecting tissues from oxidative damage and contributing to the regulation of inflammation. Recently, an unprocessed form of Hp, namely zonulin, has attracted attention for its potential role as a biomarker [140]. As an acute phase protein, Hp tends to increase in response to stress conditions. These preliminary results suggest that increased Hp levels could be considered a nonspecific biomarker of GBM [141]. Zonulin, on the other hand, has been identified exclusively in the plasma of GBM patients, suggesting a possible role in tumor progression, particularly in the processes of invasiveness and vascularization. These data suggest that the set of Hp proteoforms could be employed as a panel of biomarkers for GBM: levels of α and β chains could indicate the presence of neoplasia in a nonspecific manner, while zonulin could be a specific marker for GBM [123,140,141,142].

#### 3.2.3. Serum

In a study conducted by Popescu et al. [125] serum samples from 35 patients (14 women and 21 men) with stage IV glioblastoma multiforme were analyzed and compared with 30 healthy controls, identifying CXCL4, S100A8, and S100A9. Although these proteins have previously been associated with various types of cancer [143], they are also known as inflammatory factors [144,145].

Of particular interest is the study conducted by Clavreul et al. [146] which identified the differences in protein abundance in tumor and serum samples from patients with IDH-wildtype glioblastoma, distinguishing between short-term survivors (STS) and long-term survivors (LTS) [147,148,149]. Three tumor proteins (AHSP, FABP7, and TJAP1) were downregulated in the STS group and were not identified in the serum proteome of patients, while 26 serum proteins were upregulated in the STS group; of these, 23 proteins were also identified in the tumor proteome, but were expressed similarly in both the STS and LTS groups. Analysis of the three tumor specific proteins of interest indicated that they were associated with fatty acid transport, Golgi organization, and hemoglobin metabolic processes, respectively. The 26 serum proteins of interest were associated with different biological processes: cellular detoxification of oxidants, cellular homeostasis, regulation of reactive oxygen species metabolism, aging, purine ribonucleotide metabolism, generation of precursor metabolites, VEGFA-VEGFR2 signaling, and IL-18 signaling. In particular, in serum, two proteins were of particular interest: MDH1 and RNH1. MDH1, a central enzyme in metabolic processes such as glycolysis and glutaminolysis, was upregulated in STS patients. This protein could support tumor metabolic reprogramming by reducing oxidative stress and contributing to the Warburg effect [146].

Several studies have isolated Glioma-Associated Stromal Cells (GASCs) from the peritumoral microenvironment of glioblastoma, which had phenotypic and functional properties similar to those of mesenchymal stem cells and CAF [146,150]. These GASCs, which have prognostic value in glioma, can undergo metabolic reprogramming and induce metabolic reprogramming in glioblastoma cells via MDH1.

Kun et al. [127] demonstrated that high expression levels of five driver genes, including RNH1, were associated with poor prognosis in glioblastoma patients. High levels of RNH1 in STS serum could therefore arise from high production in glioblastoma cells to reduce ROS production, as hypothesized for MDH1.

#### 3.2.4. Urine and Saliva

Urine, with its relative simplicity and high sensitivity of analytical techniques, can be an effective “liquid biopsy” for continuous GBM monitoring. Saliva, on the other hand, with its disease-influenced dynamic composition, emerges as an equally promising alternative, particularly for non-invasive monitoring of progression and response to treatment. However, further studies in larger cohorts are needed to validate these approaches and establish their clinical applicability on a wider scale. Urine and saliva can be collected easily, rapidly, and non-invasively, allowing for repeated analysis over time without stressing the patient. A particularly interesting aspect concerns urinary extracellular vesicles (uEV), which contain a wide range of proteins able to reflect tumor burden and GBM progression. The use of advanced mass spectrometry techniques allowed precise quantification of proteins in uEV, improving sensitivity and accuracy compared to traditional methods [128].

An interesting study by Hallal et al. [128] identified two key biomarkers, progranulin (GRN) and prosaposin (PSAP), both associated with tumor recurrence and treatment resistance, through LC-MS/MS with DIA. In particular, proteomic analysis of uEV showed that GRN and PSAP differ in the various stages of the disease (pre-operative, post-operative, and recurrence), highlighting their potential in the surveillance of progression and therapeutic response [117]. Other biomarkers such as ALDOA and S100-A11 were confirmed in uEV and other body fluids, suggesting the potential of urine as a diagnostic source for disease monitoring. uEV also presents proteins associated with treatment resistance, such as ITM2B, which is identified as a significant biomarker for GBM recurrence [151]. Another interesting group of proteins includes the subunits of the TRiC complex (TCP1, CCT2, CCT3, CCT4, CCT6A, CCT7, and CCT8), which were found in significantly increased amounts in preoperative samples from GBM patients [117,118].

Saliva has also emerged as an interesting source of biomarkers for GBM, especially due to the presence of small EVs that reflect the phenotypic composition of tumor cells [152,153,154,155]. A detailed proteomic analysis was performed by Müller et al. [129], which allowed for the identification of 507 proteins in salivary EVs from GBM patients. Of these, 238 proteins were present exclusively in preoperative samples, 215 proteins were detected both before and after treatment, and 54 were found only in postoperative samples. These data suggest a significant heterogeneity in the proteomic content of salivary EVs, which could be used to differentiate disease stages and monitor treatment efficacy [153]. Analysis of DEPs (also referred to as DAPs—differentially abundant proteins) revealed that some proteins, such as ALDOA, 1433E, and TM11B, are associated with unfavorable prognostic outcomes. ALDOA, an enzyme involved in glycolysis, was linked to cellular proliferation and treatment resistance in various cancers, suggesting that its overexpression in GBM cells could indicate a negative prognosis [156,157]. Similarly, the protein 1433E (YWHAE) is crucial for cell cycle regulation and signaling pathways and has been associated with astrocytomas [156,158,159]. The analysis also highlighted the overregulation of proteins such as C3, a complement system component, and PPIA, involved in protein folding and intracellular trafficking, both central to tumor progression [160,161,162]. Unique proteins identified in salivary EVs, including immunoglobulins and proteins related to the TGF-β signaling pathway, strengthen the idea that saliva may contain exclusive biomarkers for GBM. Furthermore, the enriched molecular pathways, such as those associated with the immune system, complement cascade, and iron metabolism, indicate the key role of saliva in assessing tumor progression. These findings suggest that saliva could not only be used to diagnose GBM but also to monitor therapeutic responses and recurrence risk [129,153].

## 4. Proteomics and Biobanking as Cornerstones of Translational Medicine

To achieve high response rates and maximum reliability of results, the proteomics approach requires high-quality biological samples, collected and stored according to rigorous and standardized protocols. Biobanks offer this guarantee, ensuring the availability of well-characterized materials associated with accurate clinical data and metadata. However, to maximize the effectiveness and impact of research, it is essential to promote the creation of a translational proteomics network that includes biobanks with shared governance and uniform procedures for cataloging and storing samples. This coordinated infrastructure represents even more of a prerequisite for generating reliable and usable data, suitable for multicenter and integrated studies. Samples collected in structured biobanks reduce pre-analytical and technical variables, contributing to greater experimental robustness and reproducibility of results. Process standardization and complete traceability ensure consistency and quality, crucial elements for the validation of biomarkers and the development of new therapies. Furthermore, the desired integration of proteomic data with those from other omic sciences—genomics, metabolomics, transcriptomics—represents a fundamental step to build a solid translational network, capable of producing knowledge applicable to the development of innovative therapies for complex diseases, such as highly invasive tumors like GBMs.

The collection, storage, and use of human biological samples and related data represent the first and fundamental step in the path of translational research. In this context, it is essential to distinguish between different types of biological material storage according to the purpose of use. In a nutshell, a study-oriented collection has a specific purpose within a defined time frame, and a biorepository has a corporate purpose that often does not provide access to third parties; meanwhile, a research biobank guarantees both scientific future purposes and fair access. Below it is reported the definitions of these three main models based on the national and international consensus and regulatory frameworks.

**Figure 5:**
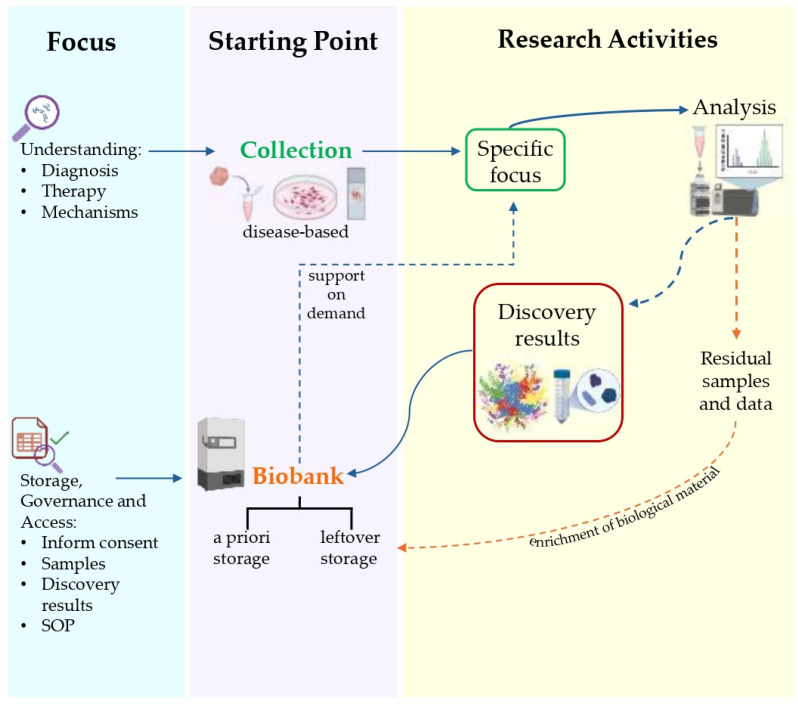
Outline of the workflow in biobank-based biomedical research, illustrating the steps from sample collection and storage to analysis, result discovery, and the reuse of residual materials for new studies.

- “Study-oriented human biological material Collections”—Collection, storage, and use of human biological materials and related data finalized to a specific project, generally oriented by pathology according to research or clinical protocols and participant’s previous expressed consent. In the protocol as in the specific consent, the start and end of the collection and use are declared, at the end of which the samples must be destroyed or biobanked, based on the further consent to biobanking expressed [163].
- “Biorepository”—A facility that collects, catalogs, and stores samples of biological material, such as urine, blood, tissue, cells, DNA, RNA, and protein, from humans, animals, or plants for laboratory research (https://www.cancer.gov/publications/dictionaries/cancer-terms/def/biorepository, accessed on 3 April 2025). In for-profit contexts, the biorepository is intended for exclusive, corporate use.
- “Research Biobank”—A legal entity, or part of a legal entity, formally established at a public or private institution; a non-profit. The research biobank as a service structure, at the service of the scientific communities, is the guarantor of the principles, rights, and processes that constitute biobanking for future research purposes. In full compliance with the informed consent/assent to research biobanking expressed and the rights of the participants involved, the biobank guarantees and manages, according to proven quality standards, the stable and continuous collection, conservation, use, and access of human biological materials, and/or related and derived data, for research. The sharing of biobanked samples/data, as well as results, is the cornerstone of all the activity of a research biobank (Figure 5) [164,165,166,167].

Of note, older collection refers to organized human biological materials originally obtained for different purposes, and subsequently stored and used for research without the explicit consent of the participants, thus not complying with current ethical and legal requirements.

Over the past decades, increasing attention to ethical and legal principles has prompted a progressive shift from the use of historical collections, often lacking informed consent, to the development of structured and compliant approaches to the acquisition and management of biological materials. This evolution reflects the growing need for transparency, standardization, and participant protection, and has laid the foundation for modern biobanking practices based on rigorous scientific, ethical, and procedural frameworks.

Initially, it takes the form of a simple collection process, in which tissues, fluids or other biological materials are obtained from research participants or patients according to standardized protocols. However, this phase does not end with the mere act of collection: it requires a careful acquisition procedure to ensure the integrity of the biological material and its suitability for subsequent analysis.

With increasing complexity, the collection process becomes part of a broader methodological framework, in which validation, preservation, and traceability criteria assume a crucial role. Each sample is accompanied by detailed data, including clinical, molecular, and environmental information, essential to contextualize experimental results and ensure reproducibility.

At a more advanced level, sample collection evolves into biobanking, a highly regulated practice based on ethical, legal, and scientific principles as well as on IT-based anonymization/cryptography and harmonized quality standards.

Research biobanks are recognized as vital components of translational research infrastructure [168]. They play a fundamental role in collecting, processing, storing, using, and distributing biological samples and related data. Biobanks serve as the cornerstone of translational medicine, understood as an inter-disciplinary branch of the biomedical field supported by three main pillars: bench side, bedside, and community [169]. Reliable biological samples are crucial for confirming and validating both basic and preclinical research [170].

Biobanks, as guarantors of the principles, rights and processes, have a strategic position in promoting the reliability and reproducibility of future research data, as well as in supporting responsible research and innovation [171,172].

**Figure 6:**
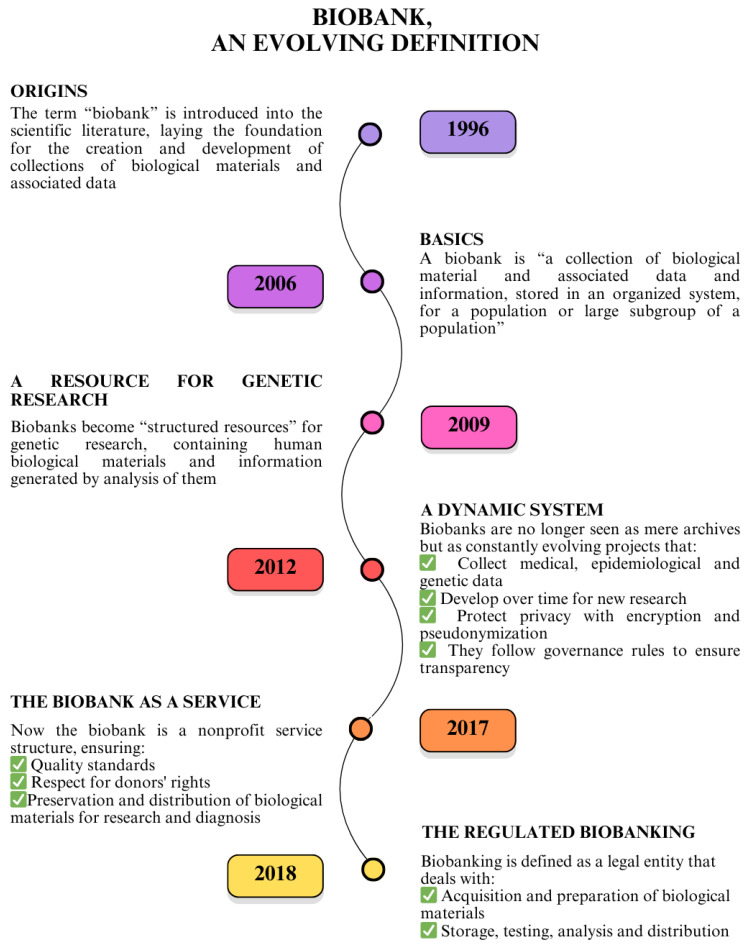
Evolution of the biobank concept. This infographic illustrates the progressive transformation from simple biological material collections to complex, regulated biobanking infrastructures integrating scientific research, data protection, and public health frameworks.

The term “biobank” first appeared in the literature in 1996 [173]. In 2009, the OECD proposed the following consensus definition of the biobank and genetic dataset: “structured resources that can be used for genetic research purposes and that include (a) human biological materials and/or information generated by the analysis thereof; and (b) associated extended information” [174]. This was in conjunction with the first steps of the Pan-European Research Infrastructure for Biobanking and Biomolecular Resources—BBMRI, which in 2013 was formally recognized as an European Research Infrastructure Consortium (ERIC), i.e., a permanent legal entity under European law (www.bbmri-eric.eu) (Figure 6).

Over the past 15 years, the ethical–regulatory framework, along with a global consensus on quality standards for research biobanks, has been consolidated through European and international soft laws, international standards, governmental bodies, and national and international infrastructures; for example, the International Guidelines for Health-related Research Involving Humans of the Council for International Organizations of Medical Sciences (CIOMS)-WHO, Guidelines 11 and 12, and other relevant frameworks [175].

In 2017, the Italian BBMRI community (https://www.bbmri.it/ accessed on 24 March 2025) helped define the role of the biobank as a custodian, as well as a third party, with the following consensus [176], to arrive in 2018 at a global International Organization for Standardization (ISO) standard specifically dedicated to biobanks and biobanking for research, ISO 20387:2018 defines a Biobank as “a legal entity or part of a legal entity that performs Biobanking” and the term Biobanking as “the process of acquisition and preservation, together with some or all of the activities related to the collection, preparation, storage, testing, analysis and distribution of defined biological material and related information and data” [177].

A quality management system (QMS) governs and supervises daily biobanking procedures, ongoing training for operators, corrective actions for non-conformities, personnel safety, and the maintenance of instruments [171]. Biobanks also address ethical, regulatory, and privacy issues [178,179] in an era where the distinctions between ethical principles, research, and clinical care are often challenging to define [180].

Furthermore, only well-preserved frozen biospecimens are ideal for evaluating the genome, transcriptome, and proteome [181].

Finally, for its development, fair and open access is required for researchers to substantial collections of human biological samples, which are well annotated [182], respect FAIR (Findable, Accessible, Interoperable, Reusable)-Health principles [183] in compliance with the ethical–legal–social requirements, which are fundamental to ensuring the accessibility and usability of biological material in the long term.

The BBMRI-ERIC is one of the largest distributed European Research Infrastructures in the “Health and Food” domain as defined by the European Strategy Forum on research Infrastructure (ESFRI, https://www.esfri.eu/, accessed on 24 March 2025). The primary goal of BBMRI-ERIC is to optimize and facilitate pan-European biomedical research by providing access to biobanked samples and related data. This is achieved through sharing and harmonizing good practices and dedicated services, including ethical, legal, and social services, as well as IT and quality services. BBMRI-ERIC offers unique access to samples and data from over 400 biobanks that are formally part of the infrastructure, all of which comply with ethical, legal, and quality standards. Two specific digital tools, “Locator” and “Negotiator,” support this process. The Locator helps researchers find valid samples and related data stored in biobanks, while the Negotiator facilitates interactions between researchers and biobanks when requesting samples and data.

Moreover, it is recommended that Standard Operating Procedures (SOPs) be aligned, wherever possible, with the procedures specified in the WHO/IARC guidelines for biological resource centers dedicated to cancer research. Finally, an economic sustainability plan will be outlined to ensure the long-term continuity of the network.

## 5. Perspectives for a Glioblastoma Translational Network

In the present overview, we report the state of the art in proteomics for glioblastoma, highlighting a potential increase in its incidence, approximately ±20% over the last 10 years. This trend underscores the need to strengthen a translational research network dedicated to glioblastoma, leveraging innovative approaches such as proteomics.

The multidisciplinary experts involved in this paper represent the preliminary nucleus of a Translational Proteomics Network (TPN), which aims to classify and manage more than one thousand GBM-affected subjects according to standardized biobanking procedures. These samples will be analyzed using state-of-the-art proteomic technologies. The TPN’s primary goals include the identification of early diagnostic biomarkers, potential therapeutic targets for GBM, and tools for effective disease monitoring.

The network should involve hospitals treating glioblastoma patients, as well as biobanking experts who can manage samples and associated data within their own facilities or according to their areas of expertise. Additionally, proteomics can benefit from integration with other omic approaches, such as genomics and metabolomics, to create a comprehensive picture of GBM.

The translational network can take advantage of recent advancements in biobanking, including shared governance, coordination, and standardized cataloging, while addressing ethical, legal, and social implications. These include tools such as informed consents, Material Transfer Agreements (MTAs), Data Transfer Agreements (DTAs), and well-defined policies for the use, access, and return of results.

However, the greatest synergistic potential lies in the integration of clinical data, enabling a complementary convergence between molecular findings (e.g., genes, proteins, and metabolites) and phenotypic characteristics (e.g., clinical and imaging data). This approach reflects the conceptual transition from quantity (molecular expression) to quality (observable phenotype), echoing Hegel’s dialectical principle as expressed in his Science of Logic (1812) [184,185].

To ensure secure data handling, a shared pseudonymization process must be implemented, using persistent unique identifiers and a standardized IT system for managing biobanks within the Glioblastoma Translational Network.

The TPN’s overarching goal is to incorporate proteomic evaluation into the framework of translational research, utilizing the infrastructure of high-quality biobanks while fostering a cooperative approach that aligns with European and international standards. The network also aims to facilitate the sharing of biospecimens and associated data, standardizing their collection and storage, and supporting the development of focused pilot projects that will enhance the effectiveness of GBM research.

Moreover, it is crucial to involve civil society, individuals and companies, in the development of P4 medicine (predictive, preventive, personalized, and participatory medicine) [186]. This approach is further strengthened by advances in systems biology and medicine, which examine health and disease through a global, integrative approach. This contributes to the “One Health” concept, which emphasizes the human–environment interaction [187]. Finally, the recent addition of a fifth ‘P’ (P5 medicine), which addresses psycho-cognitive aspects and the quality of life, underscores the active role of patients in both research and treatment processes [188].

## Figures and Tables

**Table 1:** Proteomics approaches applied to the analysis of brain tissue and cells, with identified candidate biomarkers and associated pathways. References provided in the final column of the table support the methodological approaches described.

Species	Sample Type	Proteomic Approach	Biomarkers Identified	Functional Relevance	Ref.
Human	Tissue	Labeling (TMT; iTRAQ)	ASAH1, GPNMB MMP9, TIMP1, Fibulins EGFR, NPM1, RKIP HNRNPK, ELAVL1, NOVA1	Sphingolipid metabolism and ferroptosis Immune microenvironment Tumor progression, migration and angiogenesis Signaling growth and resistance to therapy Controlling gene expression in GBM	[60,61,62,63,64,65,66]
		No labeling: LC-MS/MS (LFQ; DDA; DIA)	YAP1, SOX2, PP1γ EGFR, FN1, PTEN, BRAF FN1, TNC, ICAM1, GAGs HIF1α, IDH1, OXPHOS, Cholesterol, HSPD1, Granzyme A, STAT3, CHI3L1 RPS5, SF3B2, HMGB2 ASAH1, p21-p53-RB, ERCC2, POLD1	Proliferation and survival Tumor growth and migration ECM regulation and cell adhesion Tumor metabolism and hypoxia Immune response and immunosuppression RNA processing and splicing Cell survival, apoptosis, DNA damage	[67,68,69,70,71,72,73,74,75,76,77,78,79,80,81]
	Cells	No labeling: LC-MS/MS (LFQ; DDA; DIA)	ADAM10, ADAM15, COL6A1, COL1A2, COL6A3, TIMPs, Fibulin-2/-5/-7 STAT1, STAT2, OAS, IFIT, TRIM25, PME-1, PP2A-B55α, MAPKAPK2, RIPK1 CSE1L, TAZ, Importin α5, WWTR1, RAD51	ECM regulation and tumor progression IFN signaling Sensitivity to oxidative stress Apoptosis, DNA damage	[82,83,84,85,86,87,88,89,90,91,92,93,94,95,96,97,98,99,100,101,102,103,104,105,106,107,108,109,110,111]
Animal	Tissue	Labeling: iTRAQ	ILF2, CCT7, CCT4, RPL10A, MSN, PRPS1, TFRC, APEX1	Early brain development Primary formation of the neural tube, Regulation of neuronal differentiation, Synaptic transmission, Regulation of the nervous system Regulation of cell survival and tumor proliferation Mechanisms of drug resistance and chemosensitivity	[112]
	Cells	No labeling: LC-MS/MS (DDA; DIA)	CaMK2, BCAS1, FBXO2, INF2, PRPS2 CD9, CD81, Nono, Gja1	Tumor growth, microenvironment Response to hypoxia, glycosaminoglycan biosynthesis Integrin-mediated signaling pathways, regulation of TGFβ pathways	[94,113]

**Table 2:** Proteomic approaches applied to the analysis of biofluids, with identified candidate biomarkers and related molecular information. References in the final column support the methodologies and findings reported.

Sample Type	Proteomic Approach	Biomarkers Identified	Functional Relevance	Ref.
Cerebrospinal Fluid	LC-MS/MS (DDA)	CHI3L1 GFAP, GAP43, SERPIN3, APOE, FGA, FGB, FGG, F2	Tumor aggressiveness BBB disruption Synaptogenesis Coagulation Angiogenesis LXR/RXR Activation pathway Stemness, Immune modulation	[41,121]
Cystic Fluid	LC-MS/MS (DDA)	Albumin, Haptoglobin, Fibrinogen, Transferrin, Prostaglandin D2 synthase, IgG, IgA, IgM, S100B, GFAP	Cell adhesion, angiogenesis and cytoskeleton Acute Inflammatory Response Immunomodulation	[122]
Plasma	LC-MS/MS (DDA)	ASAH1, SYNM, GPNMB, VWF, Hp (α, β chains), zonulin	Tumor progression, invasiveness and vascularization Neutrophil involvement Pro-angiogenic processes and pro-thrombotic response Oxidative stress protection Inflammation regulation and homeostasis	[60,123,124]
Serum	LC-MS/MS (DIA)	CXCL4 (PF4), S100A8, S100A9, MDH1, RNH1, FABP7, TJAP1, AHSP	Inflammation Ros metabolism Nucleotide metabolism Metabolic reprogramming Cellular homeostasis Lipid metabolism and transport VEGF and IL-18 signaling	[125,126,127]
Urine	LC-MS/MS (DIA)	GRN, PSAP, ALDOA, S100A11, ITM2B, TCP1, CCT2, CCT3, CCT4, CCT6A, CCT7, CCT8	Proteostasis and protein folding Metabolic reprogramming Tumor progression Stress response	[128]
Saliva	LC-MS/MS (DIA)	ALDOA, 14-3-3ε (YWHAE), TM11B, C3, PPIA, TGF-β-related proteins	Cellular proliferation Cell cycle Signaling regulation Complement system activation Protein folding and trafficking TGF-β signaling Immune response Iron metabolism	[129]

## Abbreviations

GBM	Glioblastoma Multiforme
CNS	Central Nervous System
CSF	Cerebrospinal Fluid
TCGA	The Cancer Genome Atlas
TMZ	Temozolomide
BBB	Blood–Brain Barrier
GSC	Glioblastoma Stem Cells
NCI	National Cancer Institute
ISS	Istituto Superiore di Sanità
LC-MS/MS	Liquid Chromatography–Tandem Mass Spectrometry
DIA	Data-Independent Acquisition
ECM	Extracellular Matrix
SWATH-MS	Sequential Window Acquisition of All Theoretical Mass Spectra
TMT	Tandem Mass Tag
CUSA	Cavitron Ultrasonic Surgical Aspirator
LFQ	Label-Free Quantification
DDA	Data-Dependent Acquisition
DEPs	Differentially Expressed Proteins
iTRAQ	Isobaric Tags for Relative and Absolute Quantitation
EVs	Extracellular Vesicles
GASCs	Glioma-Associated Stromal Cells
uEV	Urinary Extracellular Vesicles
OECD	Organisation for Economic Co-operation and Development
BBMRI	Biobanking and Biomolecular Resources Research Infrastructure
ERIC	European Research Infrastructure Consortium
CIOMS	Council for International Organizations of Medical Sciences
ISO	International Organization for Standardization
QMS	Quality Management System
FAIR	Findability, Accessibility, Interoperability, and Reusability
ESFRI	European Strategy Forum on Research Infrastructures
SOPs	Standard Operating Procedures
TPN	Translational Proteomics Network
MTA	Material Transfer Agreement
DTA	Data Transfer Agreement
